# A comprehensive risk factor analysis using association rules in people with diabetic kidney disease

**DOI:** 10.1038/s41598-023-38811-5

**Published:** 2023-07-20

**Authors:** Tadashi Toyama, Miho Shimizu, Taihei Yamaguchi, Hidekazu Kurita, Tetsurou Morita, Megumi Oshima, Shinji Kitajima, Akinori Hara, Norihiko Sakai, Atsushi Hashiba, Takuzo Takayama, Atsushi Tajima, Kengo Furuichi, Takashi Wada, Yasunori Iwata

**Affiliations:** 1 Department of Nephrology and Laboratory Medicine, Kanazawa University, Kanazawa, Japan; 2 Innovative Clinical Research Center, Kanazawa University, Kanazawa, Japan; 3 Life Science Business Office, Corporate Technology Planning Division, Toshiba Corporation, Tokyo, Japan; 4 Insurance Solutions Department, ICT Solutions Division, Toshiba Digital Solutions Corporation, Kawasaki, Japan; 5 Department of Hygiene and Public Health, Kanazawa University, Kanazawa, Japan; 6 Kanazawa Medical Association, Kanazawa, Japan; 7 Frontier Science and Social Co-Creation Initiative, Kanazawa University, Kanazawa, Japan; 8 Department of Bioinformatics and Genomics, Graduate School of Advanced Preventive Medical Sciences, Kanazawa University, Kanazawa, Japan; 9 Department of Nephrology, Kanazawa Medical University School of Medicine, Uchinada, Japan

**Keywords:** Prognostic markers, Chronic kidney disease, Machine learning

## Abstract

Association rule is a transparent machine learning method expected to share information about risks for chronic kidney disease (CKD) among diabetic patients, but its findings in clinical data are limited. We used the association rule to evaluate the risk for kidney disease in General and Worker diabetic cohorts. The absence of risk factors was examined for association with stable kidney function and worsening kidney function. A confidence value was used as an index of association, and a lift of > 1 was considered significant. Analyses were applied for individuals stratified by KDIGO’s (Kidney Disease: Improving Global Outcomes) CKD risk categories. A General cohort of 4935 with a mean age of 66.7 years and a Worker cohort of 2153 with a mean age of 47.8 years were included in the analysis. Good glycemic control was significantly related to stable kidney function in low-risk categories among the General cohort, and in very-high risk categories among the Worker cohort; confidences were 0.82 and 0.77, respectively. Similar results were found with poor glycemic control and worsening kidney function; confidences of HbA1c were 0.41 and 0.27, respectively. Similarly, anemia, obesity, and hypertension showed significant relationships in the low-risk General and very-high risk Worker cohorts. Stratified risk assessment using association rules revealed the importance of the presence or absence of risk factors.

## Introduction

The increasing number of patients with diabetes is a global problem. One of its complications, diabetic kidney disease, is a known risk factor for end-stage kidney failure, cardiovascular events, and death^1,2^. In recent years, many hypoglycemic agents that have an organ-protective effect have become available^3^. However, risk assessment of each individual remains fundamental to the appropriate treatment of diabetic kidney disease.

Shared decision-making between healthcare providers and patients has become increasingly important in recent years^4^. Although a patient’s current risk status is a basic decision-making criterion, indicators of risk severity, such as odds ratios and hazard ratios, are generally difficult to apply in clinical practice because their methodology is not transparent to patients.

Association rules are a machine learning method used in market analysis^5^. In medical research, association rules can be applied to the relationship between risk factors and outcomes. As the results of association rules are easy to interpret, this method could be widely used to share the risks of kidney disease between patients and healthcare providers. To be used in clinical practice, the results need to be consistent with previous evidence, but few studies using association rules have been conducted. Therefore, the purpose of this study was to conduct a comprehensive risk factor analysis and kidney outcomes in patients with diabetes using association rules.

## Methods

### Study participants

In this study, two populations with diabetes were separately analyzed. One was a general diabetic population cohort (General cohort), and the other was a worker-with-diabetes cohort, comprised of people insured by the Toshiba Health Insurance Society (Worker cohort). In both cohorts, people were classified as having diabetes mellitus by the following criteria: glycated hemoglobin (HbA1c) ≥ 6.5%, fasting plasma glucose ≥ 7.0 mmol/L (≥ 126 mg/dL), or treatment of diabetes mellitus^6^. The General cohort consisted of adults who underwent annual health examinations in Kanazawa, Ishikawa, Japan, between 1999 and 2018; adults aged ≥ 40 years who were not covered by company insurance were eligible to undergo the health examinations. The Worker cohort consisted of employees of a Japanese company (Toshiba Corporation), and information from annual health examinations from 2010 to 2016 was used. Both cohorts were eligible for analysis if they had serum creatinine levels measured and had at least one follow-up visit during the observation period. There was no upper age limit for the subjects. Those who refused to participate, did not have baseline information on risk factors or eGFR, or were not followed up for 5 years were excluded from the analysis. Baseline was defined as the oldest year in a data series in which eGFR is measured and then followed for at least 5 years.

### Measurement of risk factors

Risk factors recorded in the health examination were used in the analysis. The following risk factors were assessed: eGFR, urinary protein, glycohemoglobin (HbA1c), hemoglobin, aspartate aminotransferase (AST), alanine aminotransferase (ALT), γ-glutamyltransferase (GGT), total cholesterol, triglyceride, high-density lipoprotein (HDL) cholesterol, low-density lipoprotein (LDL) cholesterol, systolic blood pressure, diastolic blood pressure, body mass index (BMI), diabetic retinopathy, and 2-year decrease in eGFR from baseline.

From the serum creatinine level measured by the enzymatic method, eGFR was calculated using the formula for the Japanese population^7^. Urine was collected randomly during the day and measured by dipstick and classified as negative/trace or ≥ 1+ (1+ corresponds to a urine protein of about 30 mg/dL). Blood pressure was measured in the sitting position after resting.

### Outcomes

The outcome of worsening kidney function was defined as a ≥ 30% decrease in eGFR from baseline during the follow-up period as a surrogate marker for end-stage kidney disease^8^. In the Worker cohort, the following status was also included in the definition: diagnosis of end-stage kidney disease, initiation of maintenance hemodialysis, and kidney transplantation. The outcome of stable kidney function was defined as not achieving the outcome of worsening kidney function during the follow-up period, which was a < 30% decrease in eGFR from baseline during the follow-up period.

### Statistical analysis

In this study, we used association rules^5,9^ to examine the relationship between risk factors and outcomes. For a comprehensive analysis, we examined the absence of risk factors and stable kidney function, as well as the presence of risk factors and worsening kidney function.

The association rule is an indicator originally used in market analysis, but it is also being applied in medical research^10^. We used the value of confidence as an indicator of the association strength between the risk factor and the outcome. Confidence_stable_ is the ratio of people with stable kidney function among those with an absence of risk factors, that is expressed by the following formula. Similarly, Confidence_worsening_ is expressed as follows. For visual description, see Appendix Fig. 1.

$${\text{Confidence}}_{{{\text{stable}}}} = \frac{{{\text{n}}_{{\text{absence of a risk factor and stable kidney function}}} }}{{{\text{n}}_{{\text{absence of a risk factor}}} }}$$

$${\text{Confidence}}_{{{\text{worsening}}}} = \frac{{n_{{\text{presence of risk factor and with worsening kidney function}}} }}{{n_{{\text{presence of a risk factor}}} }}$$

Lift indicates the significance of the relationship between X and Y. In general, a lift value greater than 1.0 indicates that X and Y are dependent on each other. The lift was used to confirm whether the association indicated by the confidence was not by chance, considering the association found in the overall data. Lift is defined by the following equation. We considered confidence to be significant when lift > 1.0^11^. For visual description, see Appendix Fig. 1.

$${\text{Lift}}_{{{\text{stable}}}} = \frac{{\frac{{{\text{n}}_{{\text{absence of a risk factor and stable kidney function}}} }}{{{\text{n}}_{{\text{absence of a risk factor}}} }}}}{{\frac{{{\text{n}}_{{\text{stable kidney function}}} }}{{{\text{n}}_{{{\text{all}}}} }}}}$$

$${\text{Lift}}_{{{\text{worsening}}}} = \frac{{\frac{{{\text{n}}_{{\text{presence of a risk factor and worsening kidney function}}} }}{{{\text{n}}_{{\text{presence of a risk factor}}} }}}}{{\frac{{{\text{n}}_{{\text{worsening of kidney function}}} }}{{{\text{n}}_{{{\text{all}}}} }}}}$$

Thresholds of the value of risk factors were set at the upper or lower 20% of the population by sex, and the presence or absence of each risk factor was classified according to the threshold. For example, individuals with systolic blood pressure values above the 80th percentile or with urinary protein ≥ 1+ were classified as having a risk factor. The criteria for determining the presence or absence of each risk factor and the values of the threshold are shown in Appendix Table 1.

Association rules are usually found on a set of multiple risk factors associated with an outcome. However, a number of discovered rules are often clinically difficult to interpret^12^. To solve the problem, we conducted a stratified analysis according to the clinically important status. The background used for stratification was (1) CKD risk categories, (2) diabetic retinopathy, and (3) eGFR decline rate over 2 years. CKD risk categories are based on KDIGO’s (Kidney Disease: Improving Global Outcomes) risk categories^13^, with albuminuria replaced by dipstick proteinuria (Appendix Fig. 2). Analysis was performed for overall participants as well as participants stratification by (1), (1) and (2), as well as (1), (2), and (3).

All analyses were performed using Stata/MP statistical software (version 17; StataCorp LP, College Station, TX, USA). The graphs were plotted using the ggplot2 package (version 3.4.2) in R (version 4.2.2).

## Results

### Selection of participants and baseline characteristics

For the General cohort, 4935 people with diabetes met the eligibility criteria (Appendix Fig. 3A). For the Worker cohort, 2153 people with diabetes met the inclusion criteria (Appendix Fig. 3B). Table 1 shows the participants’ baseline characteristics. The General cohort had a mean age of 66.7 years, and about half of the patients were women. The mean eGFR was 75.0 mL/min/1.73 m^2^, and 11.6% of people had proteinuria ≥ 1+. The Worker cohort had a mean age of 47.8 years, and the majority were men. The mean eGFR was 80.1 mL/min/1.73 m^2^, and many participants had preserved kidney function.

**Table 1:** Baseline characteristics of the study population.

	General diabetic population cohort (General cohort)	Worker with diabetes cohort (Worker cohort)
Variable	(n = 4935)	(n = 2153)
Age (years)	66.7 ± 7.7	47.8 ± 5.5
Sex (men)	53.1%	96.2%
eGFR (mL/min/1.73 m^2^)	75.0 ± 18.3	80.1 ± 16.1
Urine protein (≥ 1+)	11.6%	29.1%
HbA1c (%)	7.4 ± 1.3	7.3 ± 1.4
Fasting plasma glucose (mmol/L)	7.7 ± 2.4	8.2 ± 2.4
Hemoglobin (g/dL)	13.9 ± 1.5	15.3 ± 1.3
AST (IU/L)	28.1 ± 15.7	30.5 ± 18.9
ALT (IU/L)	28.9 ± 22.6	41.7 ± 32.1
GGT (IU/L)	50.2 ± 67.4	72.7 ± 75.1
Total cholesterol (mg/dL)	208.2 ± 37.4	214.7 ± 39.5
Triglyceride (mg/dL)	150.6 ± 93.1*	179.4 ± 144.1
HDL cholesterol (mg/dL)	57.8 ± 15.8	53.4 ± 13.1
LDL cholesterol (mg/dL)	120.3 ± 34.1	130.6 ± 32.4
Systolic blood pressure (mmHg)	135.1 ± 17.7	130.2 ± 16.9
Diastolic blood pressure (mmHg)	78.3 ± 10.8	83.5 ± 11.9
Body mass index (kg/m^2^)	24.1 ± 3.4	26.7 ± 4.0

Continuous variables are expressed as mean ± standard deviation, or median (25th and 75th percentiles). Categorical variables are expressed as numbers (percentages).

*AST* aspartate aminotransferase, *ALT* alanine aminotransferase, *eGFR* estimated glomerular filtration rate, *GGT* γ-glutamyl transferase, *HbA1c* glycated hemoglobin, *HDL* high-density lipoprotein, *LDL* low-density lipoprotein.

*n = 2894.

During the follow-up period of 5 years, 237 (4.8%) people in the General cohort achieved worsening kidney function, meaning 95.2% of people achieved stable kidney function outcomes. On the other hand, 110 (5.1%) people in the Worker cohort achieved the outcome of worsening kidney function, and 94.9% of people achieved stable kidney function outcomes.

### Association rules for the overall population

**Figure 1:**
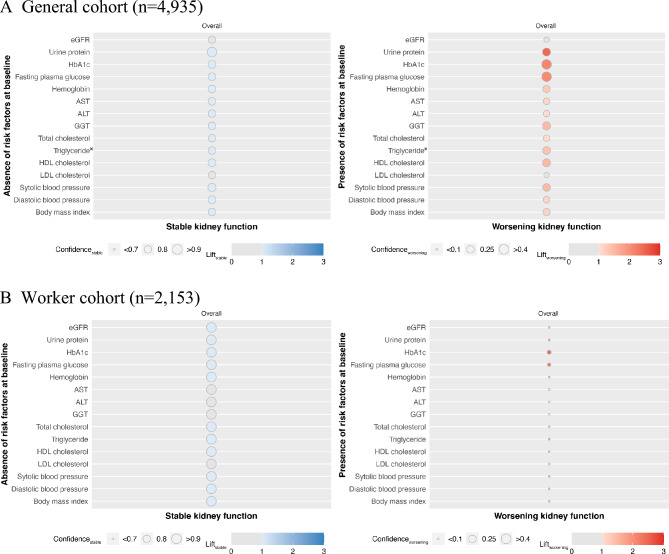
Analysis using association rules between kidney outcomes and without/with risk factors. Analysis using association rules for General cohort (**A**) and Worker cohort (**B**). The size of the circles indicates confidence, and the strength of the color indicates lift. Blue circles show the association between the absence of risk stable kidney function, and red circles show the association between the presence of risk and worsening kidney function. When the lift is ≤ 1, the circles are grayed out. *n = 2894. *AST* aspartate aminotransferase, *ALT* alanine aminotransferase, *eGFR* estimated glomerular filtration rate, *GGT* γ-glutamyl transferase, *HbA1c* glycated hemoglobin, *HDL* high-density lipoprotein, *LDL* low-density lipoprotein.

Figure 1A shows the results of the analysis using association rules for the overall General cohort. For stable kidney function, most risk factors’ confidence_stable_ was ≥ 0.7 with significance (lift_stable_ > 1). For worsening kidney function, compared to other risk factors, higher confidence_worsening_ was observed in HbA1c and fasting plasma glucose; both had confidence_worsening_ of > 0.4. The confidence_worsening_ of urine protein was 0.28 and had the highest lift_worsening_ of 2.45 among all risk factors.

Figure 1B shows the results of the association rules for the overall Worker cohort. For stable kidney function, similar to the General cohort, most risk factors’ confidence_stable_ were > 0.9 with significance. For worsening kidney function, most of the confidence_worsening_ were lower than those of General cohort. HbA1c and fasting plasma glucose were with the highest confidence_worsening_ among risk factors and with lift_worsening_ > 2.0.

### Stratified analysis using association rules by CKD risk categories

**Figure 2:**
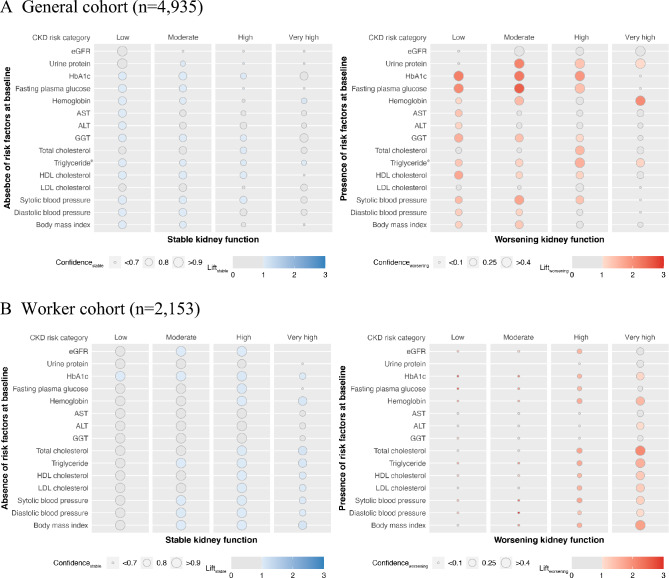
Analysis using association rules between kidney outcomes and without/with risk factors stratified by the combination of CKD risk categories. Analysis using association rules for General cohort (**A**) and Worker cohort (**B**). The size of the circles indicates confidence, and the strength of the color indicates lift. Blue circles show the association between the absence of risk stable kidney function, and red circles show the association between the presence of risk and worsening kidney function. When the lift is ≤ 1, the circles are grayed out. *n = 2894. *AST* aspartate aminotransferase, *ALT* alanine aminotransferase, *eGFR* estimated glomerular filtration rate, *GGT* γ-glutamyl transferase, *HbA1c* glycated hemoglobin, *HDL* high-density lipoprotein, *LDL* low-density lipoprotein.

Stratified analyses using association rules were conducted by CKD risk categories. Results for the General cohort are shown in Fig. 2A. For stable kidney function, in the low and moderate risk categories, high confidence_stable_ was observed for most risk factors. HbA1c and fasting plasma glucose had significant confidence_stable_ in the low-risk category (0.82 and 0.83, respectively). For worsening kidney function, in the low-risk category, HbA1c, fasting plasma glucose, GGT, HDL cholesterol, systolic blood pressure, and BMI had significantly high confidence_worsening_ (0.41, 0.37, 0.31, 0.31, 0.24, and 0.22, respectively). Few risk factors showed significance in the very-high risk category, except for hemoglobin, which had the highest confidence_worsening_ of 0.62 with a high significance (lift_worsening_ of 2.04). Triglyceride levels were significant in all CKD risk categories for both stable and worsening kidney function.

The same analyses for the Worker cohort are shown in Fig. 2B. For stable kidney function, conversely from the General cohort, significant confidence_stable_ were mainly observed in the high and very-high risk categories. In the very-high risk category, HbA1c, hemoglobin, total cholesterol, triglyceride, and BMI had significant confidence_stable_ (0.77, 0.85, 0.85, 0.84, and 0.84, respectively). For worsening kidney function, similar to the stable kidney function, risk factors with significant confidence_worsening_ were observed in the very-high risk categories (confidence_worsening_ values of hemoglobin, total cholesterol, triglyceride, HDL cholesterol, and BMI were 0.31, 0.50, 0.36, 0.31, and 0.43, respectively).

### Stratified analysis using association rules by the combination of CKD risk categories and diabetic retinopathy

**Figure 3:**
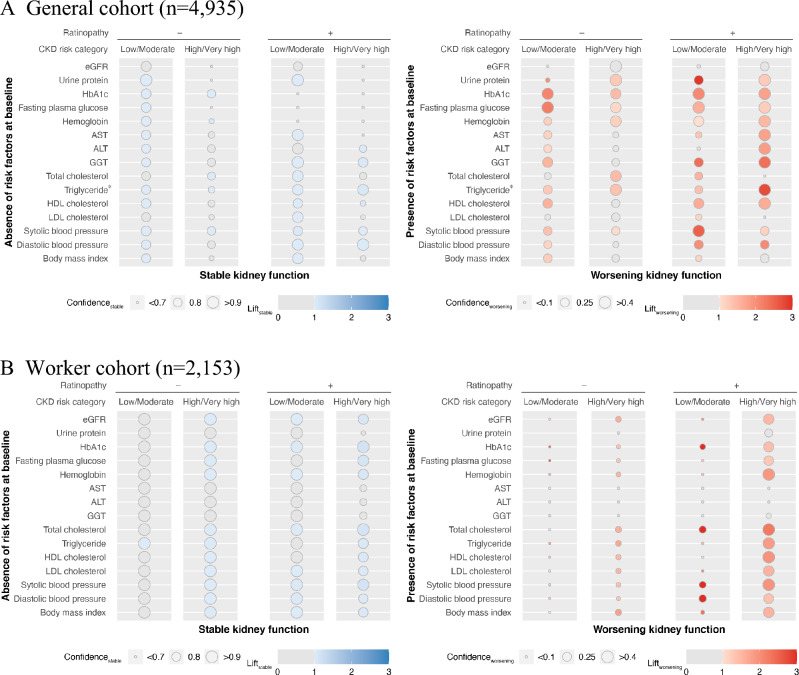
Analysis using association rules between kidney outcomes and without/with risk factors stratified by the combination of CKD risk categories and diabetic retinopathy. Analysis using association rules for General cohort (**A**) and Worker cohort (**B**). The size of the circles indicates confidence, and the strength of the color indicates lift. Blue circles show the association between the absence of risk stable kidney function, and red circles show the association between the presence of risk and worsening kidney function. When the lift is ≤ 1, the circles are grayed out. *n = 2894. *AST* aspartate aminotransferase, *ALT* alanine aminotransferase, *eGFR* estimated glomerular filtration rate, *GGT* γ-glutamyl transferase, *HbA1c* glycated hemoglobin, *HDL* high-density lipoprotein, *LDL* low-density lipoprotein.

Stratified analyses using association rules were conducted by a combination of CKD risk categories and diabetic retinopathy. Results for the General cohort are shown in Fig. 3A. For stable kidney function, confidence_stable_ of most risk factors was higher in the low/moderate risk categories than in the high/very-high risk categories, regardless of the prevalence of retinopathy. Among the low/moderate risk categories, HbA1c, fasting plasma glucose, and hemoglobin showed higher confidence_stable_ in the subgroup without retinopathy than in those with retinopathy. For worsening kidney function, like stable kidney function, almost all significant risk factors showed higher confidence_worsening_ in the low/moderate risk categories than in the high/very-high risk categories, regardless of the prevalence of retinopathy. Biomarkers of liver injury such as AST, ALT, and GGT showed significant confidence_worsening_ in the high/very-high risk categories with retinopathy. In the category, triglycerides had the highest lift_worsening_ (2.75).

The same analyses for the Worker cohort are shown in Fig. 3B. For stable kidney function, in patients without diabetic retinopathy, significant risk factors were found in the high/very-high risk categories. In patients with diabetic retinopathy, the values of confidence_stable_ were similar between high/very-high risk categories compared to the low/moderate risk categories, but higher lift_stable_ were observed in the high/very-high risk categories. For worsening kidney function, regardless of the prevalence of retinopathy, risk factors of significant higher confidence_worsening_ were found in the high/very-high risk categories. In addition, patients in the high/very-high risk categories with retinopathy showed higher confidence_worsening_ than those without retinopathy for risk factors of glycemic control, as well as lipid and blood pressure control. In the subgroup, unlike the General cohort, AST, ALT, and GGT were not significant. In the subgroup of low/moderate risk categories with diabetic retinopathy, the confidence_worsening_ of HbA1c, total cholesterol, and blood pressure were higher than other risk factors, each with high significance (lift_worsening_ > 3.0).

Additional stratification by eGFR change for two years was conducted for subgroups with data on eGFR change for the first two years of observation. The results for the General cohort are shown in Appendix Fig. 4A. For stable kidney function, in the subgroup of eGFR change ≥ − 30% and < 0%, more risk factors had significant confidence_stable_ compared to that of eGFR change ≥ 0%. No significant association was found for the subgroup of eGFR change < − 30% because no participants achieved the outcome of stable kidney function. For worsening kidney function, even with the stratification of eGFR change, significant confidence_worsening_ of risk factors—including HbA1c—were mainly found in the low/moderate risk categories. For the Worker cohort, like the General cohort, the significant confidences were mainly found in the strata of eGFR change ≥ − 30% and < 0% for both the stable and worsening kidney function (Appendix Fig. 4B).

To confirm the robustness of the results, we set the risk factor value thresholds to the upper or lower 10% of the population according to sex, similar to that done for 20% threshold, and classified the presence or absence of each risk factor according to the threshold. Similar results were obtained for both the presence and absence of risk factors (Appendix Figs. 5, 6, 7, 8).

### Ethical considerations

The study protocol was approved by the Ethics Committee of Kanazawa University and the Institutional Review Board of Toshiba Corporation (approval number AN-2021-03). For the General and Worker cohorts, opt-out model was performed with the approval of the Ethics Committee of Kanazawa University (approval number 2386 and 3670). If participants did not want to be included in the study, they provided written consent to be excluded. All analyses were performed using de-identified data. The study was carried out in accordance with the principles of the Declaration of Helsinki.

## Discussion

This study comprehensively analyzed risk factors for kidney dysfunction in people with diabetes in two cohorts using association rules. Detailed risk assessment could be performed by the association rules for stable and worsening kidney function according to the clinically important strata, including CKD risk categories, as well as the status of diabetic retinopathy. For general diabetic people, risk factors were related to kidney events in the low-risk group (low/moderate risk categories). For working diabetic people, risk factors were related to kidney events in a high-risk group (high/very-high risk categories).

### Clinical significance and study implications

Our study confirmed the associations between risk factors and loss of kidney function, which have been reported in previous studies. For example, urinary protein^1^, poor glycemic control^14^, and hypertension^15^ have been reported as risk factors for kidney dysfunction.

In general people with diabetes, our results showed that risk factors’ relationships were found in the low-risk group. Higher age was the major characteristic of this cohort. The less strict treatment goals for elderly diabetic patients with kidney disease, such as blood glucose and blood pressure, are the standard care^16^, but a study reported similar effects of glycemic control on CKD in middle-aged and elderly diabetics^17^. The detailed analysis by risk category in our study indicated the importance of risk factors among the low-risk group, even in older people.

In workers with diabetes, risk factors were associated with renal events in the high-risk group, while these associations were weaker in the low-risk group. This is most likely because the low-risk group had glomerular hyperfiltration, which is seen in the early stages of diabetic kidney disease^18^ and may have masked kidney damage. That might explain the relationships of blood glucose and blood pressure for worsening kidney function observed among the low-risk group with diabetic retinopathy. The results of this study do not deny the importance of early risk management among working-age people.

The absence of risk factors is also important for risk management. For example, good glycemic and blood pressure control were associated with stable kidney function, which is consistent with previous cohort studies^19,20^. Furthermore, similar results were found in those by CKD risk categories. The results show that absence of these risk factors is associated with stable kidney function and is in line with the guideline treatment strategy^21^.

The relationship between dyslipidemia and kidney dysfunction was shown in cases of high-risk categories and complicated retinopathy. Studies examining the relationship between dyslipidemia and kidney dysfunction have been limited^22^, but a study of advanced diabetic nephropathy in type 1 diabetes has shown results similar to ours^23^. In our study, LDL cholesterol was not associated with prognosis. This is consistent with previous observational studies in non-dialysis patients, which also reported no significant association between LDL cholesterol and renal prognosis^24^. Triglycerides, which were found to be associated with kidney events in our study, have also been associated with CKD progression and cardiovascular events^25^. LDL apheresis, a new treatment for advanced DKD patients, improves life prognosis but does not change eGFR loss^26^. Our study did not assess causality, and treatment of dyslipidemia in DKD requires further investigation.

The results of the General cohort showed that liver injury (high value of AST, ALT, or GGT) related to worsening kidney function. Studies reported that non-alcoholic fatty liver disease (NAFLD) is related to increased risk of CKD^27^, both in diabetic and non-diabetic individuals^28^. The association in the group with retinopathy has been reported in previous study^29^. This suggests that liver injury may be an important risk factor in advanced diabetic kidney disease.

Our study demonstrated the relevance of risk factors by stratifying the subjects. Although the detailed stratified analysis method is straightforward, increasing the number of strata frequently results in the "curse of dimensionality," a situation in which the number of risk factor combinations increases dramatically^30^. This is often problematic for machine learning methods. Expert or data-driven methods for identifying important features have been proposed^30^, but the interpretability of the results must still be considered.

### Strengths and limitations

One of the strengths of this study was the analysis used association rules stratified by known risk status, which made the results easy to interpret. Stratification by presence or absence of risk factors may also be helpful in sharing the risk assessment with patients. Furthermore, the General and Worker cohorts were complementary as a general population, and comparing the similarities and differences between them allowed for a better interpretation of risk factors. Conversely, this study has several limitations. First, analysis using the association rule does not allow for multivariable analysis; therefore, adjustment by covariates could not be performed. Second, this study used a combination of confidence and lift value to provide transparency. However, when using logistic regression, confidence values may be reported along with confidence intervals, which may be comprehensible in some situations. Third, there is no widely-accepted, clinically meaningful threshold of confidence value. Fourth, the threshold for presence or absence of each risk factor was set uniformly, which may not have been a clinically meaningful classification. Application of a stricter threshold may be appropriate for some risk factors. Fifth, dipstick measurement of proteinuria is known to have high false-positive rates^31^. Therefore, there is a possibility that people in this study who were classified as "presence of risk factors" based on the dipstick test may not actually have risk factors. Sixth, because the number of events of worsening kidney function in our cohorts was small in some strata, the confidence and lift could be altered by small changes in the number of events. Seventh, information on treatment was limited and could not be included in the analysis.

## Conclusion

This study used association rules for assessing the relationships between risk factors and kidney events in two diabetic cohorts. Most results were similar to previous reports, and stratified analysis allowed assessments by risk categories and retinopathy. In the future, validation studies are needed in cohorts with similar backgrounds. Analysis using the association rule is a transparent method and its efficacy for sharing CKD risk information with patients might be warranted.

## Supplementary Information

Supplementary Information.

## Data Availability

The datasets generated during and/or analyzed during the current study are not publicly available due to their containing information that could compromise the privacy of research participants but are available from the corresponding author on reasonable request.
