# Supplementary material for: A comprehensive risk factor analysis using association rules in people with diabetic kidney disease
Source: Sci Rep. 2023 Jul 20;13:11690. doi: 10.1038/s41598-023-38811-5 (PMC10359444; doi:10.1038/s41598-023-38811-5)
Supplement: Supplementary file 1 — Supplementary Information. [file 41598_2023_38811_MOESM1_ESM.docx]

A comprehensive risk factor analysis using association rules in people with diabetic kidney disease

Supplementary Material

**Appendix**

**Appendix Table 1 Definitions and values of having risk factors (General cohort)**

| **Risk factors** | **Definitions of cutoff value for presence of risk factor** | **Cutoff value of presence of risk factors** | |
| --- | --- | --- | --- |
|  |  | **Women** | **Men** |
| **eGFR (mL/min/1.73 m^2^)** | < 20^th^ percentile | < 67.0 | < 64.7 |
| **Urine protein** | ≥1+ | ≥ 1+ | ≥ 1+ |
| **HbA1c (%)** | > 80^th^ percentile | > 8.0 | > 8.0 |
| **Fasting plasma glucose (mmol/L)** | > 80^th^ percentile | > 156 | > 157 |
| **Hemoglobin (g/dL)** | < 20^th^ percentile | < 12.6 | < 13.8 |
| **AST (IU/L)** | > 80^th^ percentile | > 32 | > 34 |
| **ALT (IU/L)** | > 80^th^ percentile | > 37 | > 41 |
| **GGT (IU/L)** | > 80^th^ percentile | > 47 | > 86 |
| **Total cholesterol (mg/dL)** | > 80^th^ percentile | > 246 | > 227 |
| **Triglyceride (mg/dL)** | > 80^th^ percentile | > 170 | > 192 |
| **HDL cholesterol (mg/dL)** | < 20^th^ percentile | < 48 | < 41 |
| **LDL cholesterol (mg/dL)** | > 80^th^ percentile | > 159 | > 144 |
| **Systolic blood pressure (mmHg)** | > 80^th^ percentile | > 146 | > 146 |
| **Diastolic blood pressure (mmHg)** | > 80^th^ percentile | > 86 | > 88 |
| **Body mass index (kg/m^2^)** | > 80^th^ percentile | > 27.3 | > 26.1 |

Abbreviations: AST, aspartate aminotransferase; ALT, alanine aminotransferase; eGFR, estimated glomerular filtration rate; GGT, γ-glutamyl transferase; HbA1c, glycohemoglobin; HDL, high-density lipoprotein; LDL, low-density lipoprotein.

**Appendix Table 2 Definitions and values of having risk factors (Worker cohort)**

| **Risk factors** | **Definitions of cutoff value for presence of risk factor** | **Cutoff value of presence of risk factors** | |
| --- | --- | --- | --- |
|  |  | **Women** | **Men** |
| **eGFR (mL/min/1.73 m^2^)** | < 20^th^ percentile | < 71.0 | < 67.3 |
| **Urine protein** | ≥1+ | ≥1+ | ≥1+ |
| **HbA1c (%)** | > 80^th^ percentile | > 8.2 | > 8.2 |
| **Fasting plasma glucose (mmol/L)** | > 80^th^ percentile | > 8.8 | > 9.5 |
| **Hemoglobin (g/dL)** | < 20^th^ percentile | < 12.4 | < 14.5 |
| **AST (IU/L)** | > 80^th^ percentile | > 32 | > 38 |
| **ALT (IU/L)** | > 80^th^ percentile | > 44 | > 58 |
| **GGT (IU/L)** | > 80^th^ percentile | > 56 | > 97 |
| **Total cholesterol (mg/dL)** | > 80^th^ percentile | > 248 | > 244 |
| **Triglyceride (mg/dL)** | > 80^th^ percentile | > 182 | > 242 |
| **HDL cholesterol (mg/dL)** | < 20^th^ percentile | < 51 | < 43 |
| **LDL cholesterol (mg/dL)** | > 80^th^ percentile | > 158 | > 155 |
| **Systolic blood pressure (mmHg)** | > 80^th^ percentile | > 138 | > 141 |
| **Diastolic blood pressure (mmHg)** | > 80^th^ percentile | > 87 | > 92 |
| **Body mass index (kg/m^2^)** | > 80^th^ percentile | > 31.2 | > 30.4 |

Abbreviations: AST, aspartate aminotransferase; ALT, alanine aminotransferase; eGFR, estimated glomerular filtration rate; GGT, γ-glutamyl transferase; HbA1c, glycohemoglobin; HDL, high-density lipoprotein; LDL, low-density lipoprotein.


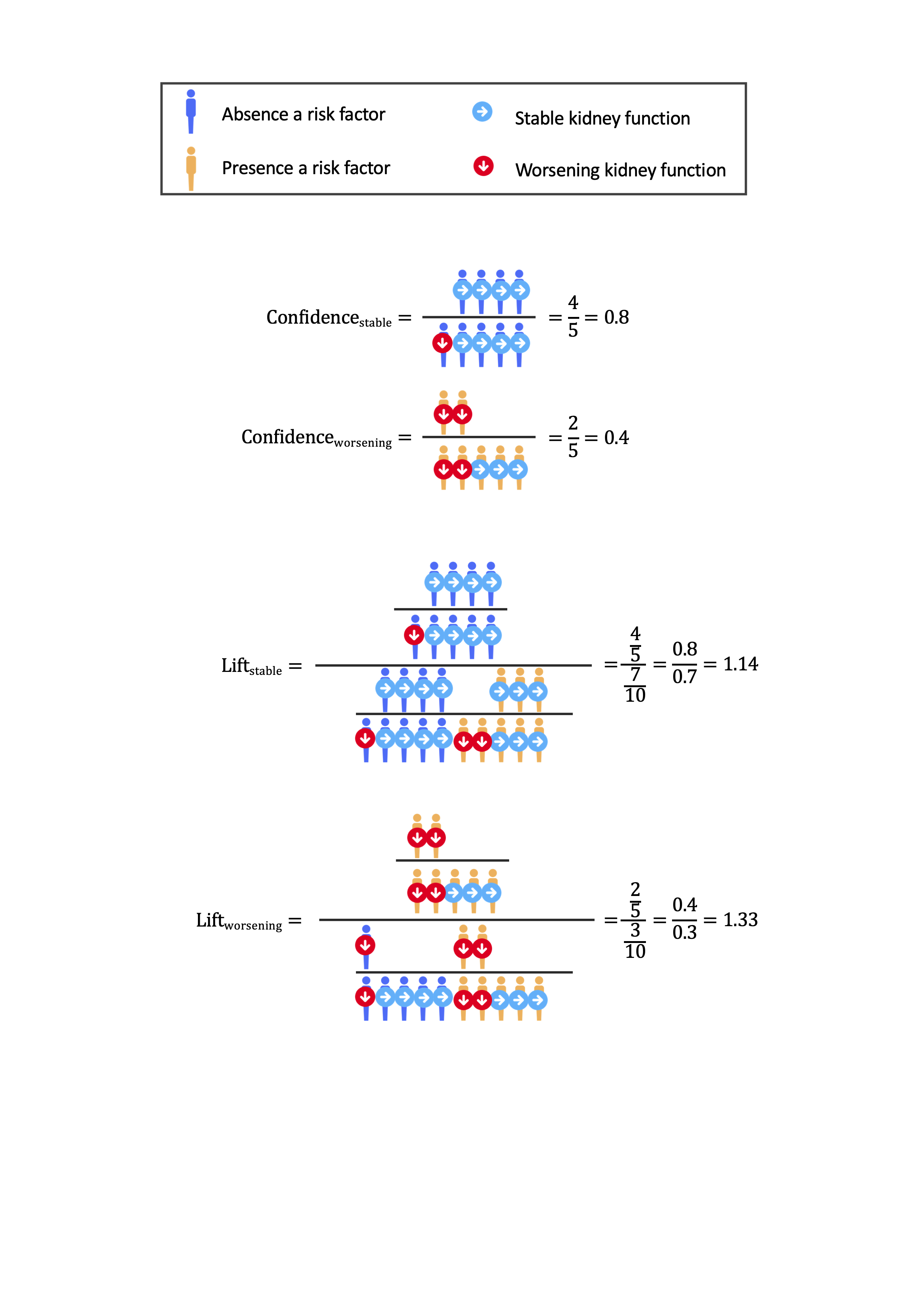


**Appendix Figure 1 Visual example of association rules, calculating confidence and lift**


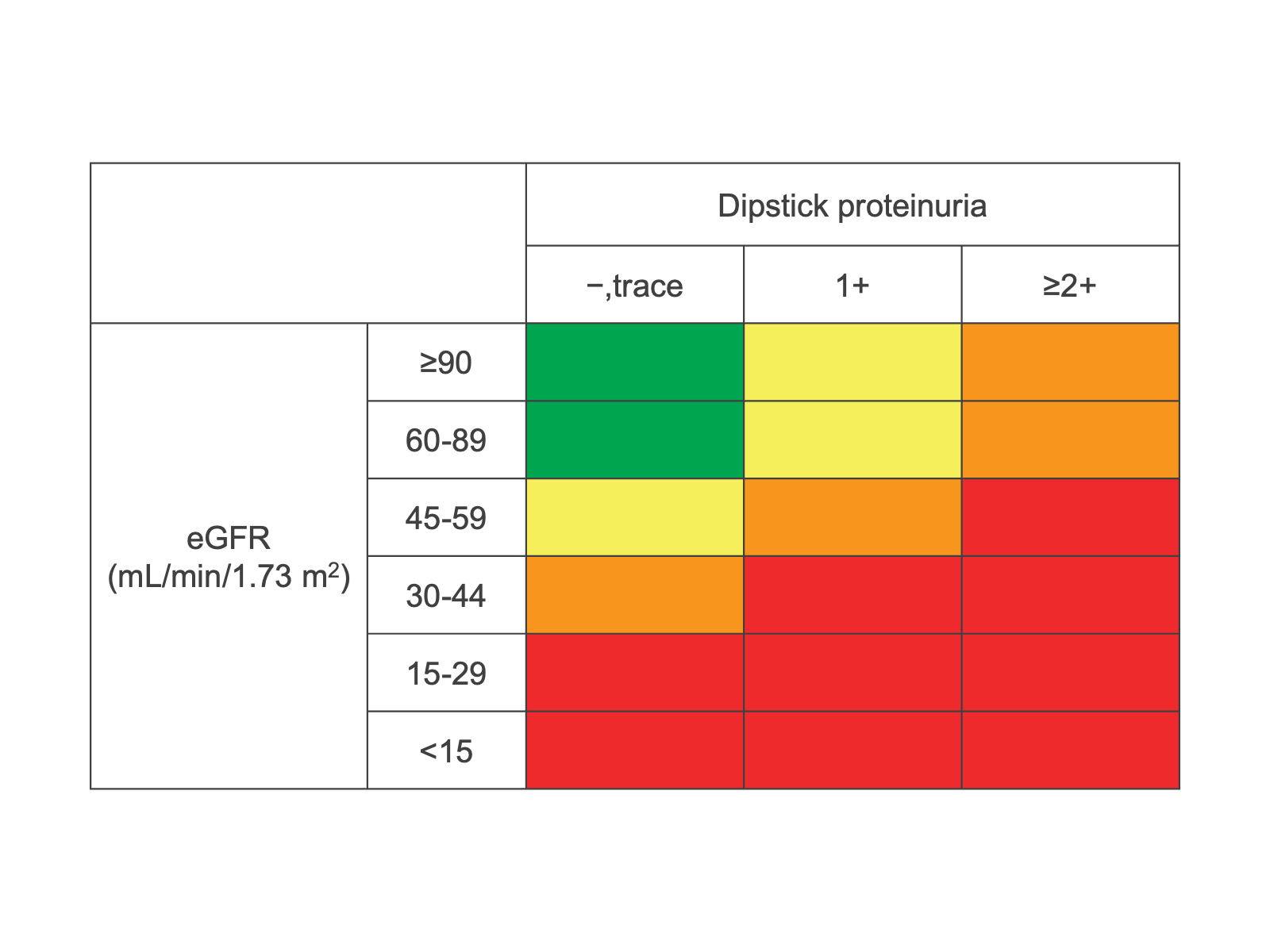


**Appendix Figure 2 CKD risk categories**

Based on KDIGO’s risk category, the risk classification was modified using dipstick proteinuria. Green: low risk category; yellow: moderate risk category; orange: high risk category; red: very-high risk category.

A General cohort


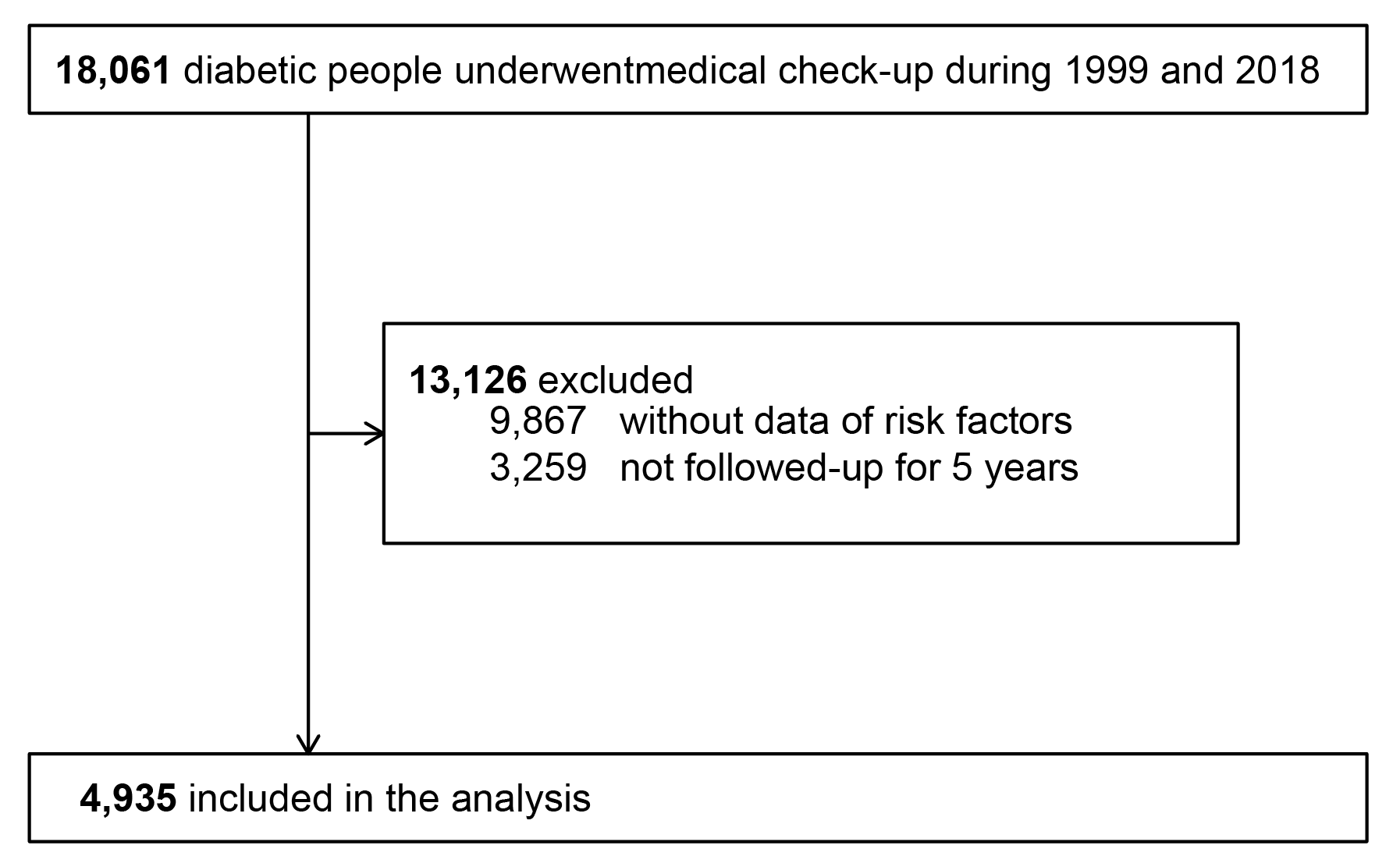


B Worker cohort


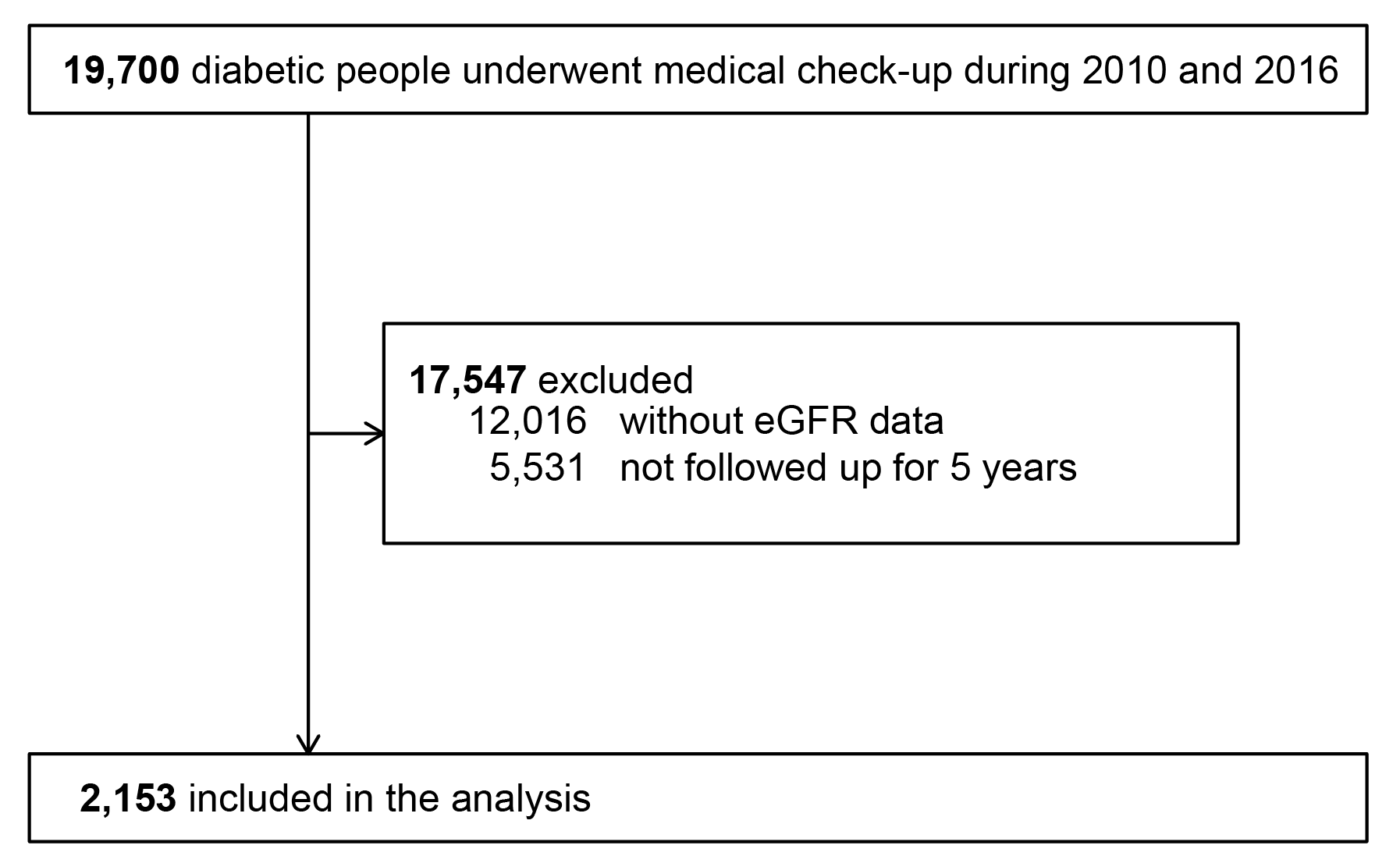


**Appendix Figure 3 Flow diagram for subject selection**

A General cohort (n=3,160)

**
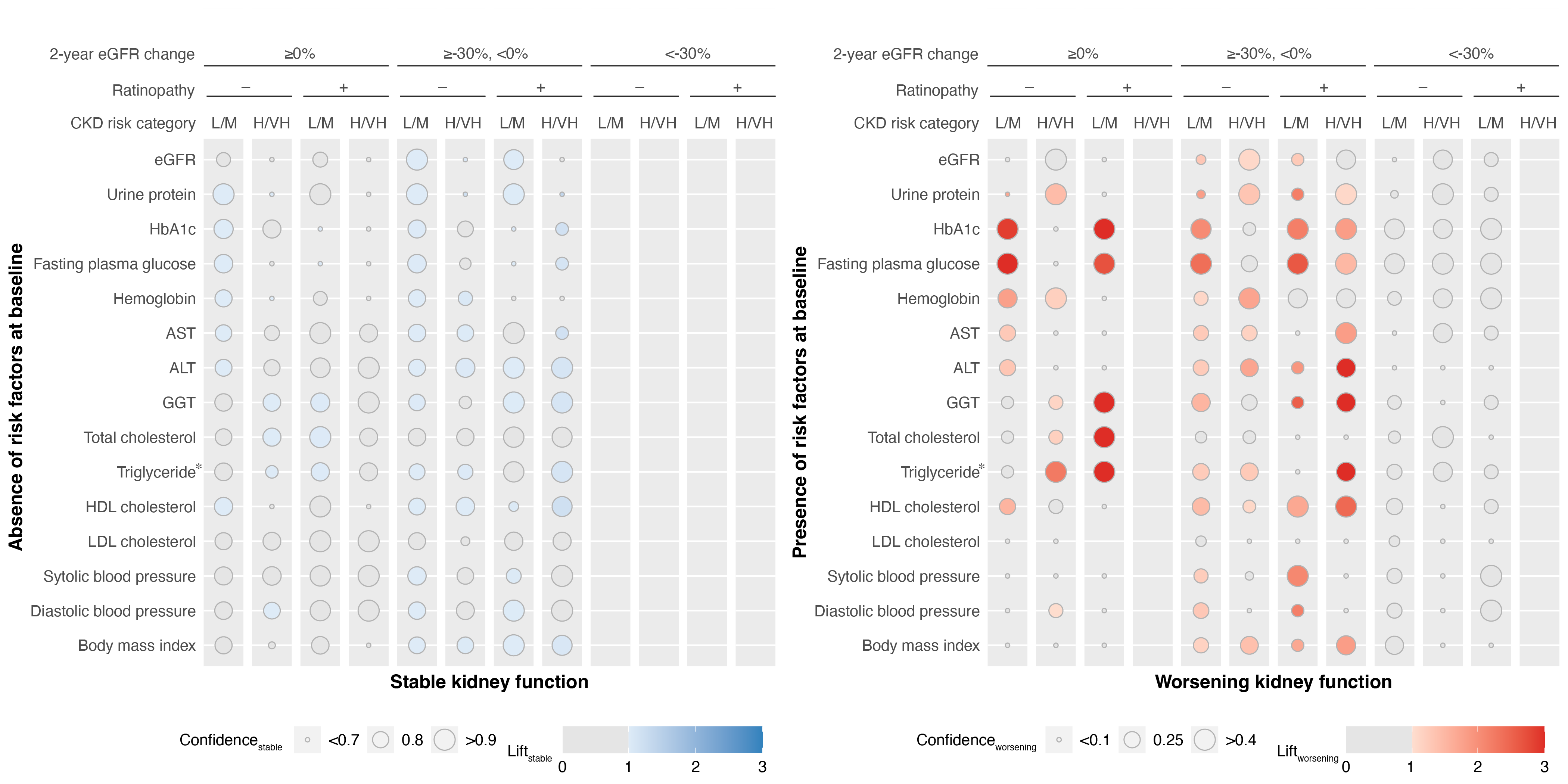
**

B Worker cohort (n=2,153)

**
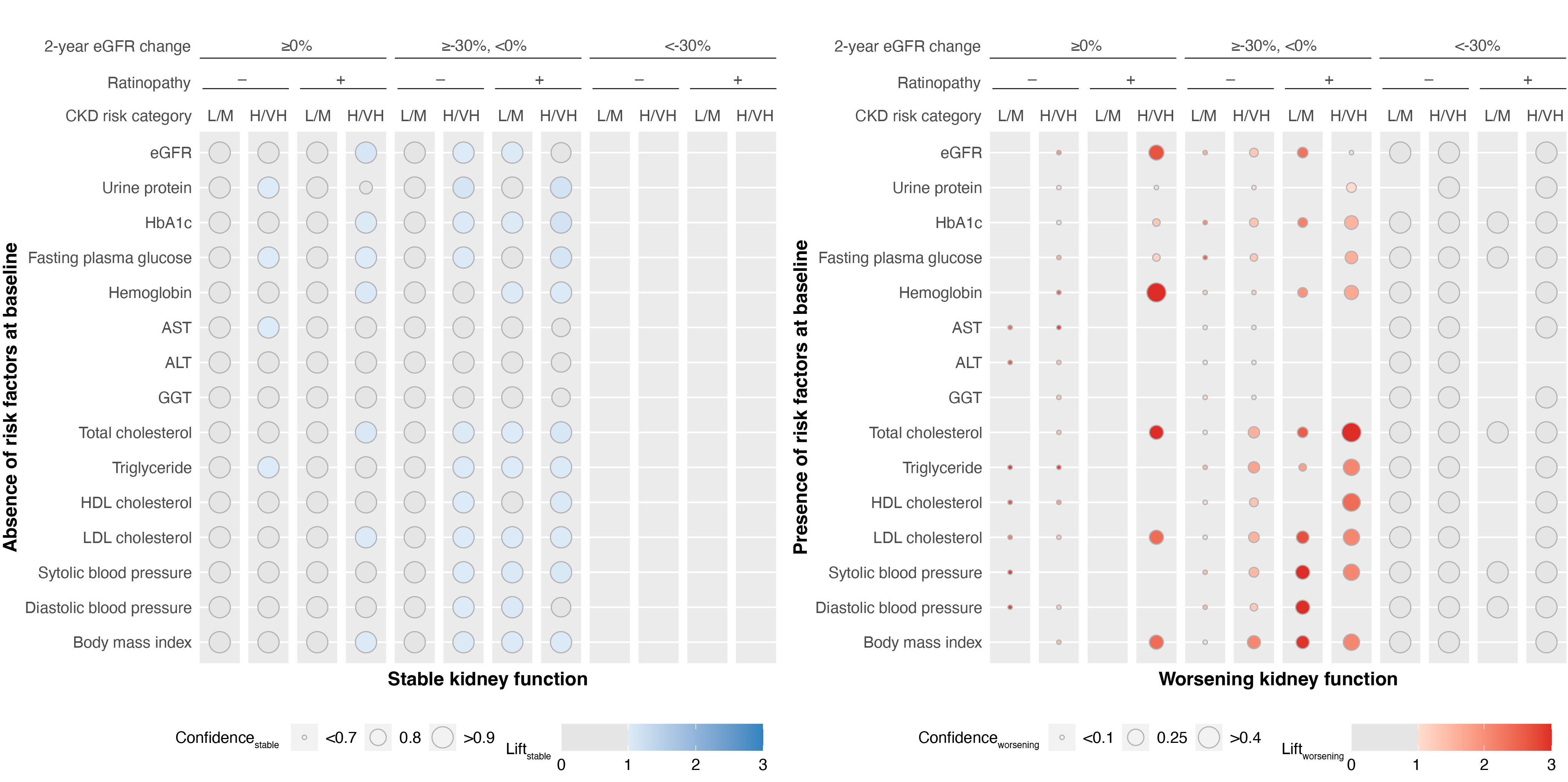
**

**Appendix Figure 4** **Analysis using association rules between kidney outcomes and without/with risk factors stratified by the combination of CKD risk categories, diabetic retinopathy, and eGFR change**

Analysis using association rules for General cohort (A) and Worker cohort (B). The size of the circles indicates confidence, and the strength of the color indicates lift. Blue circles show the association between the absence of risk and not having a kidney outcome, and red circles show the association between the presence of risk and having a kidney outcome. When the lift value is less than 1, the circles are grayed out.

Abbreviations: AST, aspartate aminotransferase; ALT, alanine aminotransferase; eGFR, estimated glomerular filtration rate; GGT, γ-glutamyl transferase; H, high; HbA1c, glycated hemoglobin; HDL, high-density lipoprotein; L, low; LDL, low-density lipoprotein; M, moderate; VH, very high.

A General cohort (n=4,935)


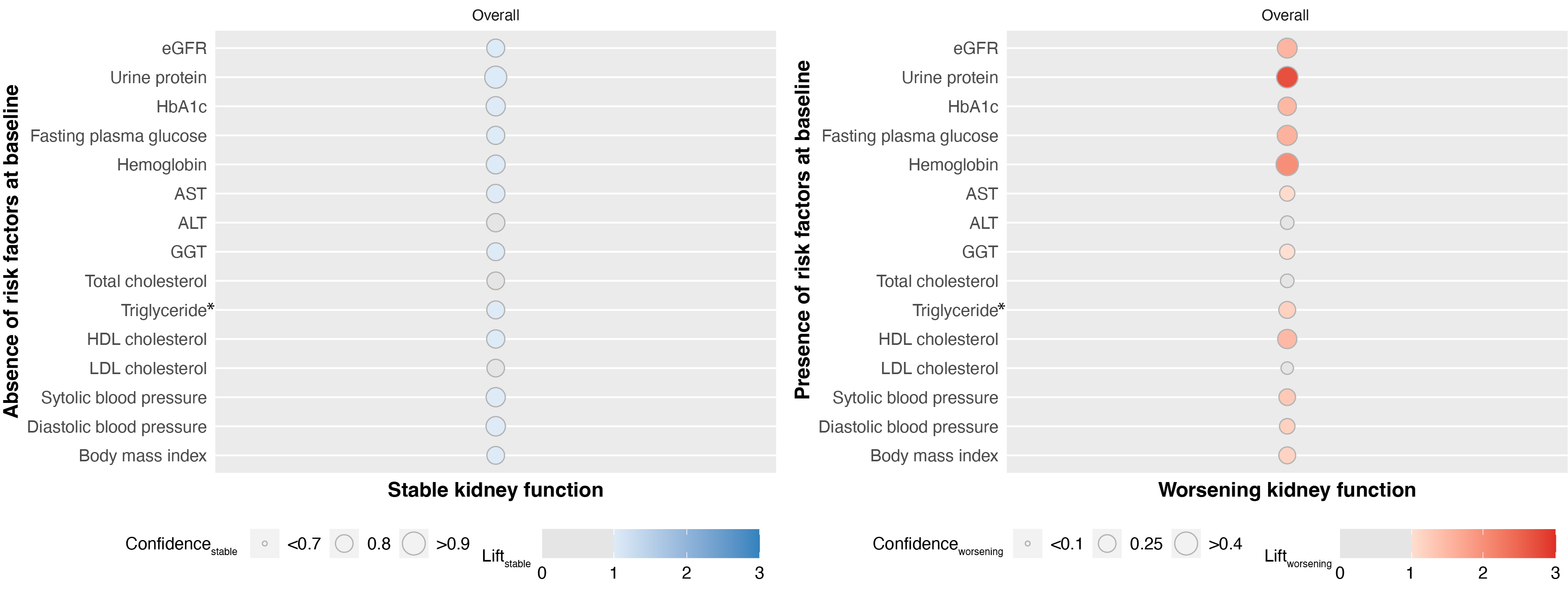


B Worker cohort (n=2,153)


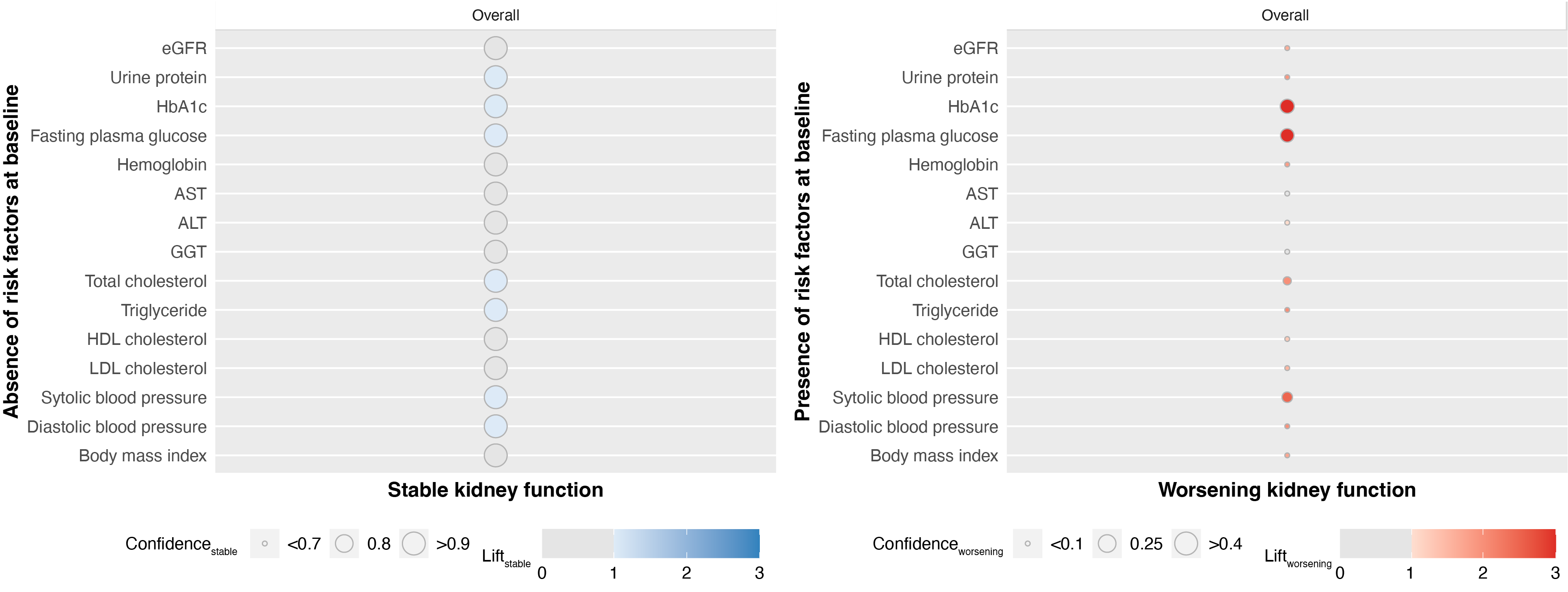


**Appendix Figure 5** **Analysis using association rules between kidney outcomes and without/with risk factors (categorized as top or bottom 10%)**

Analysis using association rules for General cohort (**A**) and Worker cohort (**B**). The size of the circles indicates confidence, and the strength of the color indicates lift. Blue circles show the association between the absence of risk stable kidney function, and red circles show the association between the presence of risk and worsening kidney function. When the lift is ≤ 1, the circles are grayed out. Thresholds of the value of risk factors were set at the upper or lower 10% of the population by sex, and the presence or absence of each risk factor was classified according to the threshold.

* n = 2,894

Abbreviations: AST, aspartate aminotransferase; ALT, alanine aminotransferase; eGFR, estimated glomerular filtration rate; GGT, γ-glutamyl transferase; HbA1c, glycated hemoglobin; HDL, high-density lipoprotein; LDL, low-density lipoprotein.

A General cohort (n=4,935)


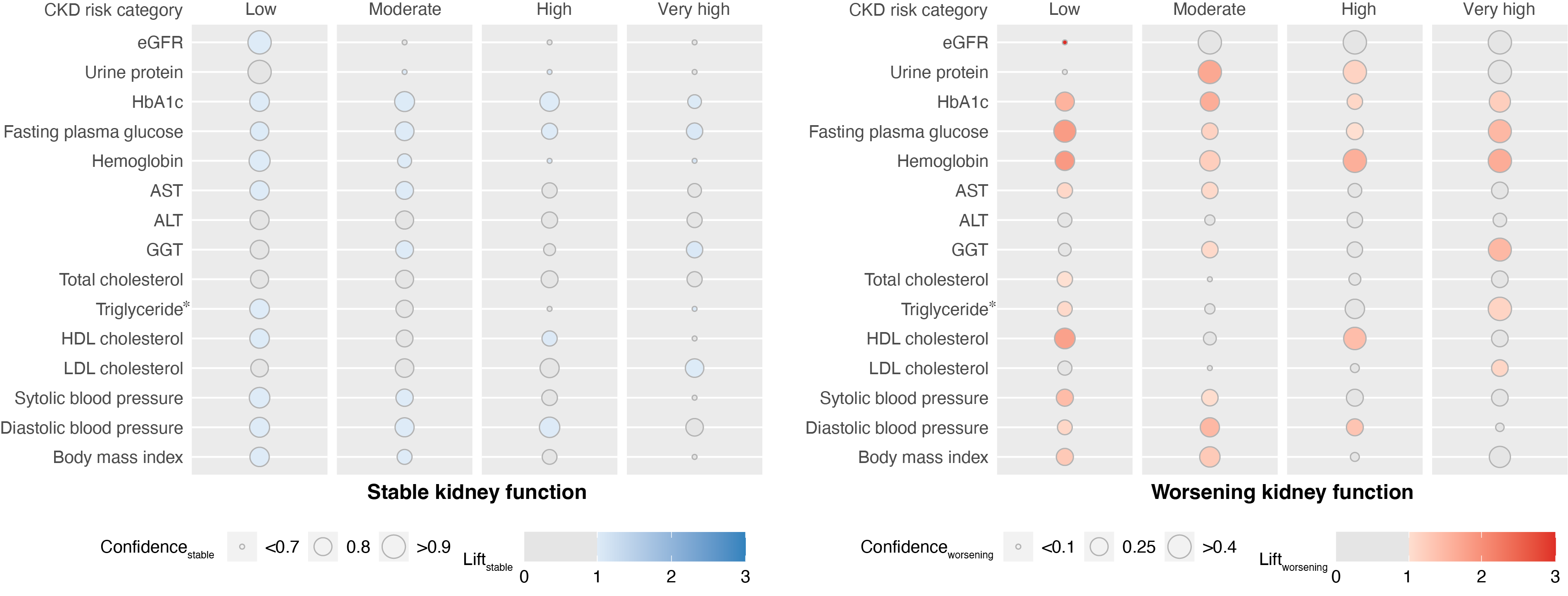


B Worker cohort (n=2,153)


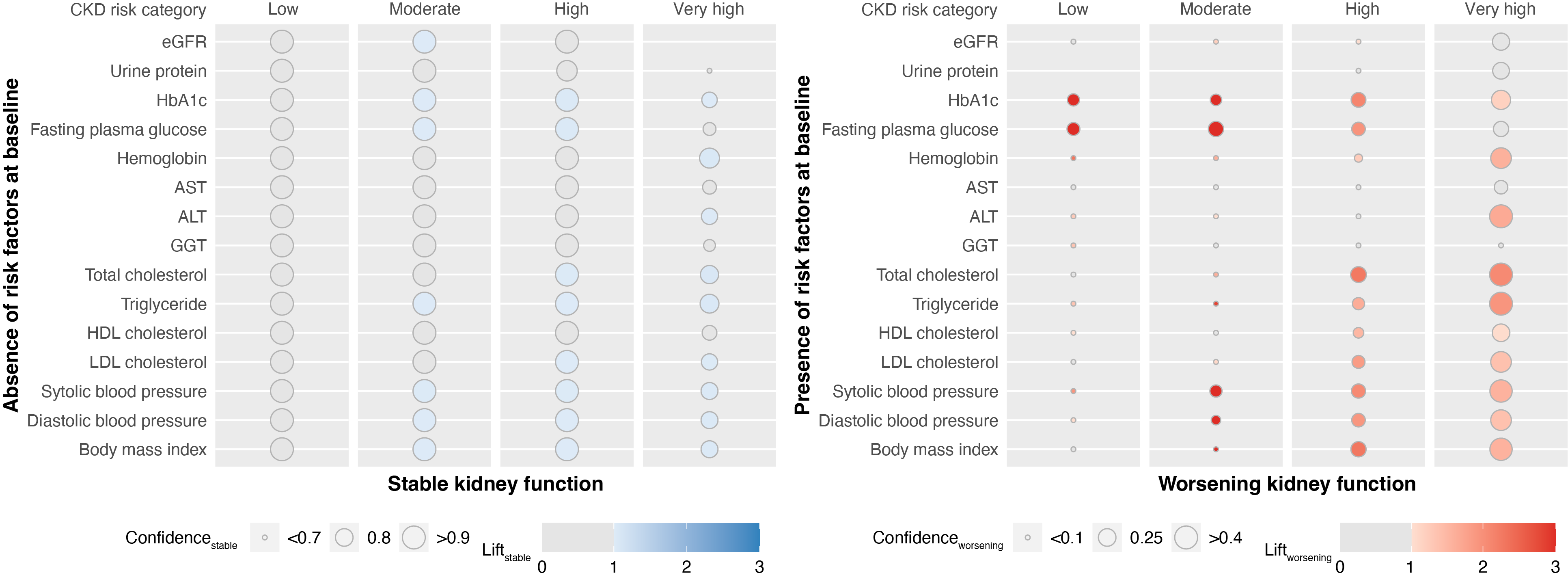


**Appendix Figure 6** **Analysis using association rules between kidney outcomes and without/with risk factors (categorized as top or bottom 10%) stratified by the combination of CKD risk categories**

Analysis using association rules for General cohort (**A**) and Worker cohort (**B**). The size of the circles indicates confidence, and the strength of the color indicates lift. Blue circles show the association between the absence of risk stable kidney function, and red circles show the association between the presence of risk and worsening kidney function. When the lift is ≤ 1, the circles are grayed out.

* n = 2,894

Abbreviations: AST, aspartate aminotransferase; ALT, alanine aminotransferase; eGFR, estimated glomerular filtration rate; GGT, γ-glutamyl transferase; HbA1c, glycated hemoglobin; HDL, high-density lipoprotein; LDL, low-density lipoprotein.

A General cohort (n=4,935)


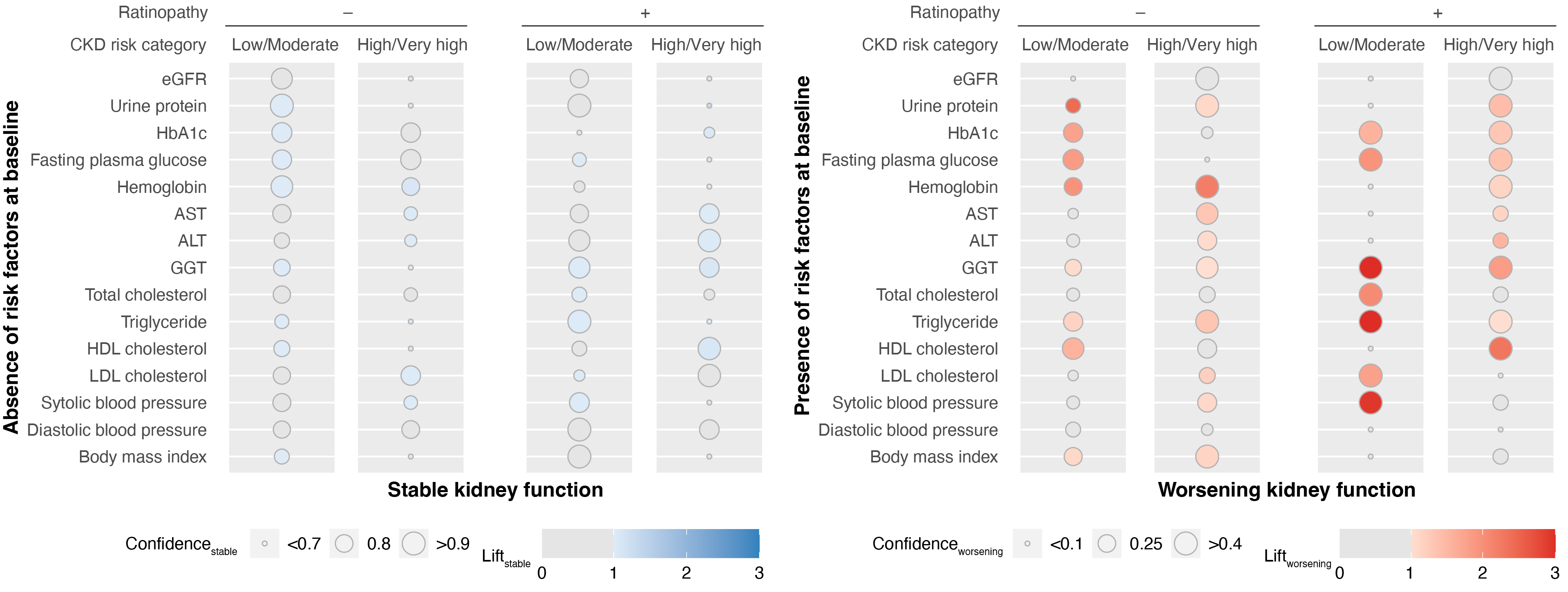


B Worker cohort (n=2,153)


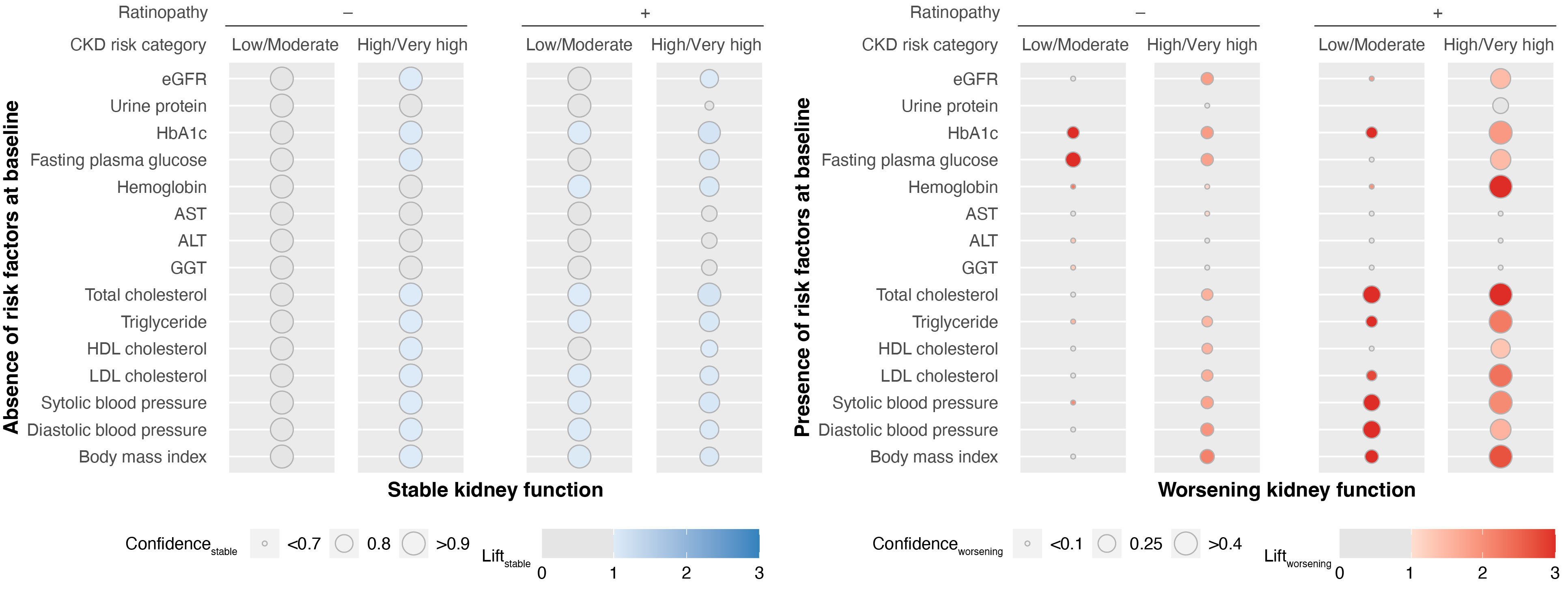


**Appendix Figure 7** **Analysis using association rules between kidney outcomes and without/with risk factors (categorized as top or bottom 10%) stratified by the combination of CKD risk categories and diabetic retinopathy**

Analysis using association rules for General cohort (**A**) and Worker cohort (**B**). The size of the circles indicates confidence, and the strength of the color indicates lift. Blue circles show the association between the absence of risk stable kidney function, and red circles show the association between the presence of risk and worsening kidney function. When the lift is ≤ 1, the circles are grayed out.

* n = 2,894

Abbreviations: AST, aspartate aminotransferase; ALT, alanine aminotransferase; eGFR, estimated glomerular filtration rate; GGT, γ-glutamyl transferase; HbA1c, glycated hemoglobin; HDL, high-density lipoprotein; LDL, low-density lipoprotein.

A General cohort (n=3,160)

**
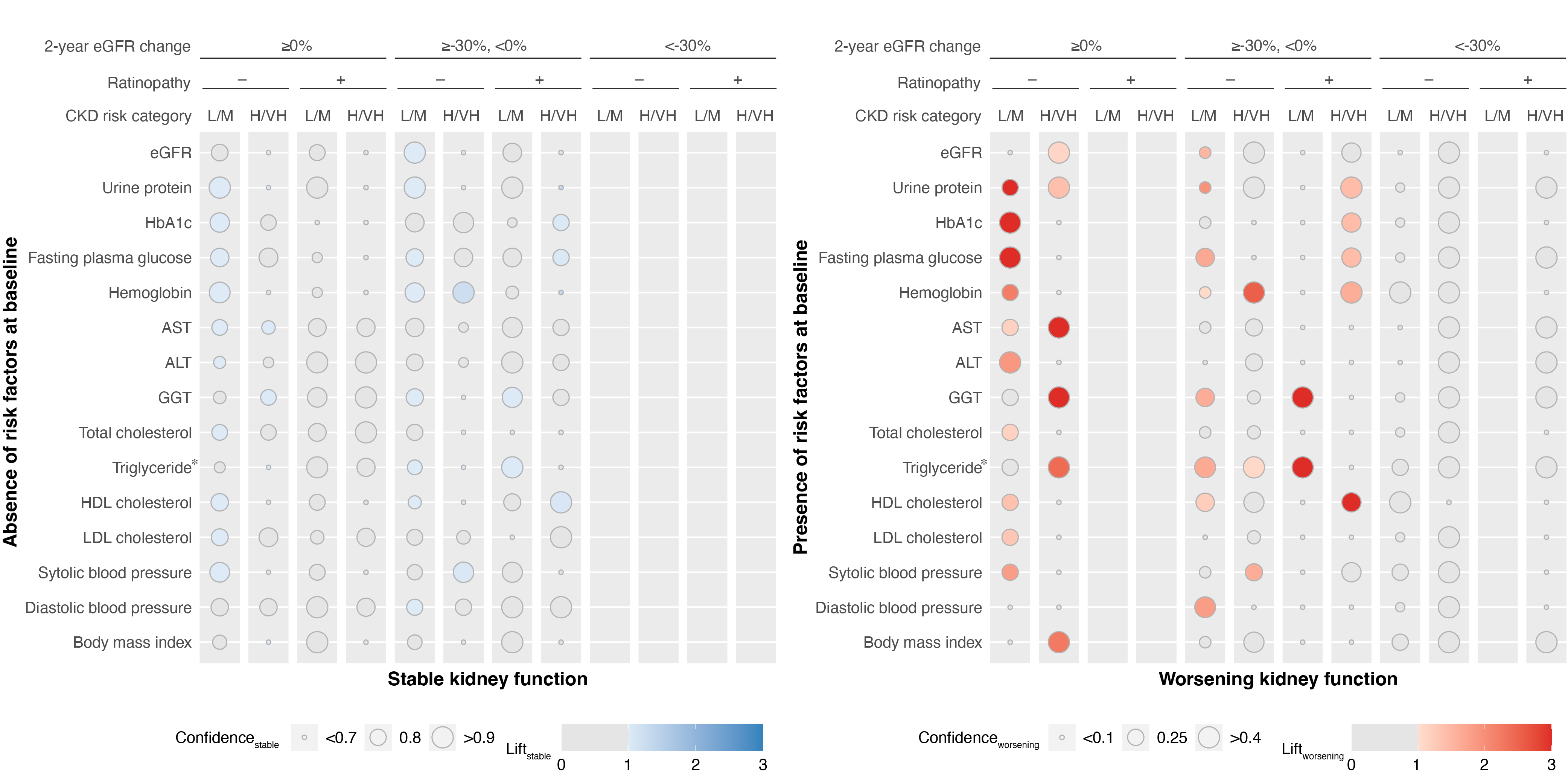
**

B Worker cohort (n=2,153)

**
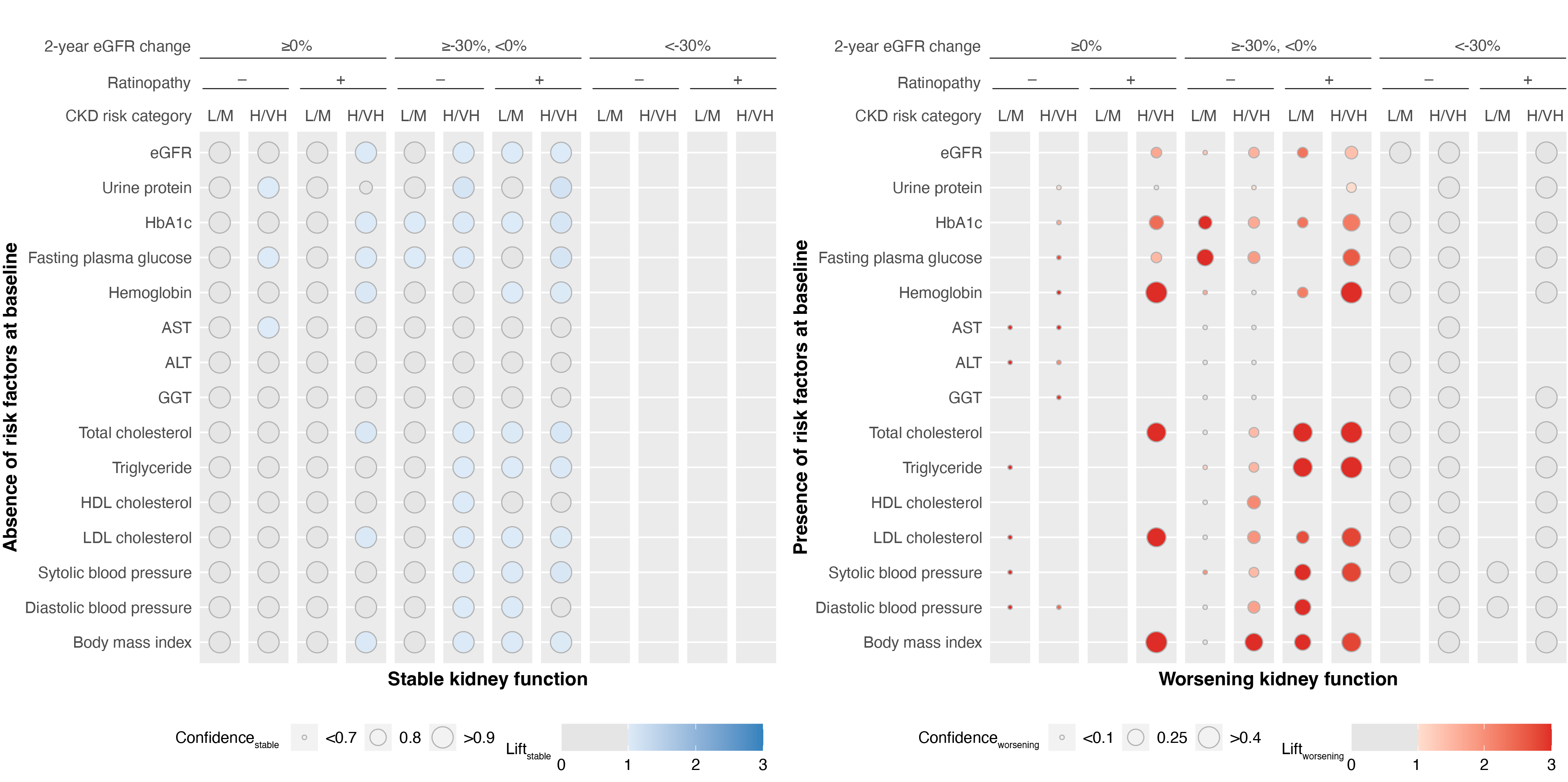
**

**Appendix Figure 8** **Analysis using association rules between kidney outcomes and without/with risk factors (categorized as top or bottom 10%) stratified by the combination of CKD risk categories, diabetic retinopathy, and eGFR change**

Analysis using association rules for General cohort (A) and Worker cohort (B). The size of the circles indicates confidence, and the strength of the color indicates lift. Blue circles show the association between the absence of risk and not having a kidney outcome, and red circles show the association between the presence of risk and having a kidney outcome. When the lift value is less than 1, the circles are grayed out.

Abbreviations: AST, aspartate aminotransferase; ALT, alanine aminotransferase; eGFR, estimated glomerular filtration rate; GGT, γ-glutamyl transferase; H, high; HbA1c, glycated hemoglobin; HDL, high-density lipoprotein; L, low; LDL, low-density lipoprotein; M, moderate; VH, very high.
